# Molecular Characterisation of *Escherichia coli* Collected From an Urban River in Johannesburg, South Africa

**DOI:** 10.1111/1758-2229.70203

**Published:** 2025-10-01

**Authors:** Luyanda Mkhize, Musa Marimani, Sanelisiwe Thinasonke Duze

**Affiliations:** ^1^ Department of Clinical Microbiology and Infectious Diseases National Health Laboratory Service and Faculty of Health Sciences, University of Witwatersrand Johannesburg South Africa; ^2^ Department of Anatomical Pathology Faculty of Health Science, University of Witwatersrand Johannesburg South Africa

**Keywords:** enteropathogenic *E. coli*, enterotoxigenic *E. coli*, *Escherichia coli*, Jukskei River, polymerase chain reaction, whole‐genome sequencing

## Abstract

Diarrheal diseases remain a significant public health concern worldwide, particularly among children under five. Surveillance is primarily focused on clinical samples. However, environmental reservoirs, particularly rivers, are increasingly recognised as critical sources of enteric pathogens. This study used whole‐genome sequencing (WGS) to characterise *Escherichia coli* isolates from the Jukskei River in Johannesburg, South Africa. Twenty‐seven *E. coli* isolates were subjected to pathotype‐specific PCR and WGS for characterisation. Diarrheagenic *E. coli* accounted for 44% (12/27) of the isolates, including enterotoxigenic, atypical enteropathogenic and a hybrid enterotoxigenic‐enteroinvasive *E. coli* . Most isolates (63%, 17/27) were O16:H48, and fimbrial typing revealed nine Fimtypes, with *fimH27* being the most prevalent at 56% (15/27). Resistance to ciprofloxacin, sulfamethoxazole and azithromycin was noted in 11% (3/27) of the isolates. The most prevalent virulence‐associated genes were *fimH, csgA, gad, terC, ompT, iss* and *yehA‐D*, associated with adhesion, invasion and stress response. Phylogroup A dominated the collection (70%, 19/27), and phylogenetic analysis revealed diversity among the river isolates. Some genetic links between human and livestock strains were noted, suggesting cross‐environmental transmission. These findings highlight the Jukskei River as a potential vehicle for *E. coli* transmission and underscore the importance of integrated surveillance across the environmental, human and animal sectors.

## Graphical abstract

*Escherichia coli* isolates from the Jukskei River in Johannesburg, South Africa, are genetically diverse, indicating multiple contamination sources and potential cross‐environmental transmission between the river, humans and livestock. Moreover, the presence of pathogenic *E. coli* (ETEC, aEPEC and ETEC/EIEC pathotypes) poses a significant public health risk.

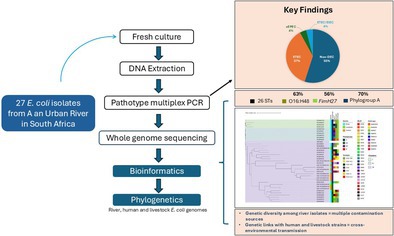

## 1 Introduction

The microorganism *Escherichia coli* ( *E. coli* ) is a commensal agent that plays a crucial role within the normal human microbiota. Although typically harmless, *E. coli* possessing virulence factors is capable of causing a wide range of intestinal and extraintestinal diseases (Riley 2020). The mode of transmission for *E. coli* varies from direct to indirect routes, such as direct exposure to infected human or animal faeces, or through the ingestion of contaminated food or water (Bolukaoto et al. 2019). Specifically, *E. coli* that causes diarrheal diseases is identified as diarrheagenic *E. coli* (DEC). DEC causes illnesses varying from diarrhoea and gastroenteritis to foodborne illnesses. Infections are more prevalent in children aged five and younger in developing countries and account for a mortality of about 2 million annually (Alfinete et al. 2022). The DEC pathotypes most commonly associated with human diseases include enteropathogenic *E. coli* (EPEC), enterohaemorrhagic *E. coli* (EHEC) or Shiga toxin‐producing *E. coli* (STEC), enteroinvasive *E. coli* (EIEC), adherent invasive *E. coli* (AIEC), enterotoxigenic *E. coli* (ETEC), diffusely adherent *E. coli* (DAEC) and the hybrid enteroaggregative *E. coli* (EAEC) (Acosta et al. 2016).

Aquatic environments such as rivers, beaches and lakes can serve as reservoirs for pathogenic bacteria, including DEC (Obi et al. 2004; Ebomah et al. 2018). In rivers, DEC poses a considerable public health risk due to its potential to contaminate freshwater sources utilised for drinking, agriculture and recreational activities (Iwu et al. 2023). A study conducted on the Buffalo River in South Africa revealed the presence of DEC strains at several access points of the river, with EHEC/STEC identified as the most common pathotype (Iwu et al. 2023). Similarly, a study that analysed water samples from three rivers in Johannesburg, South Africa, reported the presence of DEC, including ETEC, EPEC and EHEC (Bolukaoto et al. 2021).

In many developing countries, including South Africa, diarrheal diseases remain a major public health concern, particularly in immunocompromised individuals and infants (Aijuka et al. 2018). Although these diseases are notifiable and surveillance for enteric pathogens is active, surveillance efforts are primarily focused on clinical samples. However, environmental sources, especially river water, are often overlooked despite their potential role in ongoing transmission. Understanding the population structure of enteric pathogens in these environments is therefore essential for identifying potential reservoirs, tracking the spread of virulent and/or resistant strains, as well as informing public health interventions. This study aimed to characterise a collection of 27 *E. coli* isolates obtained from the Jukskei River in Johannesburg, South Africa, using whole‐genome sequencing (WGS).

## 2 Materials and Methods

### 2.1 Bacterial Strains

Twenty‐seven *E. coli* strains were selected from a collection of isolates obtained during an environmental surveillance of the Jukskei River. Briefly, water samples (*n* = 60) were collected monthly over 12 months from five sites using sterile 2 L bottles. One litre of each sample was filtered through a 0.45 μm pore‐size membrane filter (Labex, South Africa), which was transferred to a 50 mL centrifuge tube (Separations, South Africa) containing 10 mL phosphate‐buffered saline (PBS, pH 7.0; Merck, Darmstadt, Germany). Bacteria were dislodged by scraping with sterile inoculation loops and vortexing for 1 min. A 100 μL aliquot of the resulting suspension was plated on Xylose Lysine Deoxycholate (XLD) agar (Thermo Fisher Scientific, USA), and presumptive *E. coli* colonies were identified by their characteristic yellow morphology. One to two colonies per site were randomly selected, subcultured on 5% blood agar (Thermo Fisher Scientific, USA), and incubated at 37°C for 24 h. Biochemical confirmation was performed using oxidase (Merck, Darmstadt, Germany) and indole (Thermo Fisher Scientific, USA) tests, followed by molecular confirmation using polymerase chain reaction (PCR) targeting the *uidA* gene.

### 2.2 Molecular Confirmation of the *E. coli* Isolates

The *E. coli* isolates were confirmed using conventional PCR targeting the *uidA* gene. Crude DNA was extracted using the boiling method. Briefly, 400 μL of Tris‐EDTA (TE) buffer (Merck, Darmstadt, Germany) was transferred into a 1.5 mL Eppendorf tube. A loopful of culture was added to the TE buffer, vortexed and boiled at 95°C for 25 min using a heating block; thereafter, centrifuged at 12,000*g* for 3 min (Eppendorf centrifuge 5417C, Merck, NJ, USA). Following centrifugation, 20 μL of supernatant was removed and transferred added into a new 1.5 mL Eppendorf tube containing 80 μL of TE buffer. The mixture was vortexed briefly and then centrifuged at 12,000*g* for 1 min to ensure the DNA sample was collected at the bottom of the tube. The remaining pellet and supernatant from the initial centrifugation step were discarded. The resulting 100 μL of DNA samples were then kept at −20°C for further use.

The PCR reaction was performed in an Applied Biosystem MiniAMP thermal cycler (Thermo Fisher Scientific, MA, USA) in a total volume of 25 μL. The reaction consisted of 12.5 μL of PCR master mix (2×) (Thermo Fisher Scientific, MA, USA), 2 μL of each primer (Integrated DNA Technologies, IA, USA), 6.5 μL of nuclease‐free water (Thermo Fisher Scientific, MA, USA) and 2 μL of genomic DNA. The forward primer (5′‐CTGGTATCAGCGCGAAGTCT‐3′) and the reverse primer (5′‐AGCGGGTAGATATCACACTC‐3′) had an expected product size of 556 bp and were adapted from a study by Anastasi et al. (2010). The thermal cycling conditions were as follows: initial activation of 95°C for 15 min, 35 cycles of denaturation at 94°C for 45 s and annealing at 56°C for 30 s, extension at 72°C for 2 min and a final extension at 72°C for 5 min. A positive control ( *E. coli* ATCC 35218) and nuclease‐free water (no‐template control [NTC]) were included in each reaction for quality control. PCR amplicons were electrophoresed on a 2% (w/v) agarose gel prepared with 10 μL of GelRed (Thermo Fisher Scientific, MA, USA) in 1× TBE buffer. Electrophoresis was conducted at 110 V for 45 min. A 1 kb molecular weight marker (Anatech, South Africa) was used as a reference to estimate the size of the PCR products. The gels were visualised under UV transillumination, and electrophoretic images were captured using the Gel Doc EZ System (Universal Hood II Gel Doc System, Bio‐Rad, CA, USA).

## 3 Results

**FIGURE 1:**
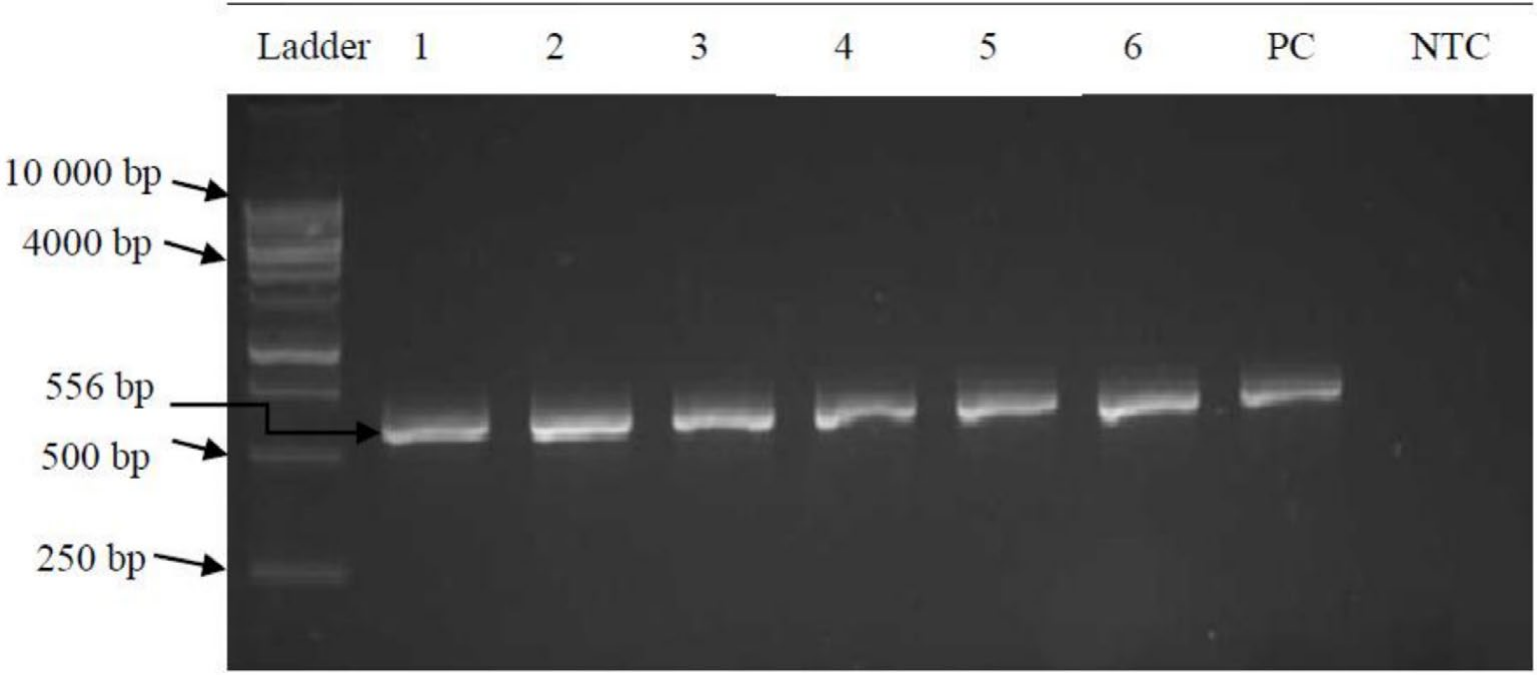
A 2% (w/v) agarose gel showing the amplification of the *uidA* gene (556 bp). A 1 kb DNA molecular weight marker was used to size the PCR products of isolates. Lanes 1, 2, 3, 4, 5 and 6 are the isolates ECBR0223, ECGI0523, ECMA0423, ECBU0523, ECBU0723 and ECDA1023, respectively. Lane 7 is the positive control ( *Escherichia coli* ATCC 35218), and Lane 8 is the NTC (nuclease‐free water).

All 27 isolates were confirmed as *E. coli* following successful amplification of the *uidA* gene by PCR (Figure 1). Moreover, 37% (10/27) of the isolates were positive for the *lt1* gene, thus classified as ETEC. Whereas one *E. coli* isolate was positive for the *eaeA* gene and was therefore classified as an aEPEC. The remaining isolates were negative for the pathotype‐specific VAGs and were thus classified as non‐DEC (Table 1).

**TABLE 1:** Pathotype‐specific VAGs detected in 27 *Escherichia coli* isolates from the Jukskei River. Presence (+) or absence (−) of target genes, as determined by five PCR assays, is shown for each isolate.

Isolate	Virulence‐associated genes											Pathotype
	*eaeA*	*stx1*	*stx2*	*rfbE*	*wbdl*	*ial*	*eagg*	*lt1*	*eaeA*	*bfpA*	*asta*	
ECBR0223	−	−	−	−	−	−	−	−	−	−	−	Non‐DEC
ECBR0423	−	−	−	−	−	−	−	−	−	−	−	Non‐DEC
ECBR0723	−	−	−	−	−	−	−	−	−	−	−	Non‐DEC
ECBR1023	−	−	−	−	−	−	−	−	−	−	−	Non‐DEC
ECBR1123	−	−	−	−	−	−	−	−	−	−	−	Non‐DEC
ECGI0223	−	−	−	−	−	−	−	−	−	−	−	Non‐DEC
ECGI0423	−	−	−	−	−	−	−	−	−	−	−	Non‐DEC
ECGI0523	−	−	−	−	−	−	−	−	−	−	−	Non‐DEC
ECGI0723	−	−	−	−	−	−	−	−	−	−	−	Non‐DEC
ECGI1023	−	−	−	−	−	−	−	−	−	−	−	Non‐DEC
ECGI1123	−	−	−	−	−	−	−	−	−	−	−	Non‐DEC
ECMA0223	−	−	−	−	−	−	−	**+**	−	−	−	ETEC
ECMA0423	−	−	−	−	−	−	−	**+**	−	−	−	ETEC
ECMA0523	−	−	−	−	−	−	−	**+**	−	−	−	ETEC
ECMA0723	**+**	−	−	−	−	−	−	**−**	**+**	−	−	aEPEC
ECMA1123	−	−	−	−	−	−	−	**+**	−	−	−	ETEC
ECBU0223	−	−	−	−	−	−	−	**+**	−	−	−	ETEC
ECBU0423	−	−	−	−	−	−	−	**+**	−	−	−	ETEC
ECBU0523	−	−	−	−	−	−	−	**+**	−	−	−	ETEC
ECBU0723	−	−	−	−	−	−	−	**+**	−	−	−	ETEC
ECBU1023	−	−	−	−	−	−	−	**+**	−	−	−	ETEC
ECBU1123	−	−	−	−	−	−	−	**+**	−	−	−	ETEC
ECDA0223	−	−	−	−	−	−	−	−	−	−	−	Non‐DEC
ECDA0423	−	−	−	−	−	−	−	−	−	−	−	Non‐DEC
ECDA0723	−	−	−	−	−	−	−	−	−	−	−	Non‐DEC
ECDA1023	−	−	−	−	−	−	−	−	−	−	−	Non‐DEC
ECDA1123	−	−	−	−	−	−	−	−	−	−	−	Non‐DEC

Abbreviations: a‐EPEC, atypical enteropathogenic *Escherichia coli* ; ETEC, enterotoxigenic *Escherichia coli* ; non‐DEC, nondiarrhegenic *Escherichia coli*.

The *E. coli* isolates were diverse, with 26 distinct STs identified. A total of three O‐antigen types and five H‐antigen types were found among the isolates. O16 was the most common O‐type, detected in 25 isolates (93%), while H48 was the predominant H‐type, present in 17 isolates (63%). Thus, the O16:H48 serotype was the most prevalent, accounting for 63% (17/27) of the isolates, followed by O16:H25 (11%, 3/27). Fimbrial typing revealed nine Fimtypes, with *fimH27* predominating (56%, 15/27), followed by *fimH34* and *fimH305* at 11% (3/27) and 7% (2/27), respectively. One *E. coli* isolate ECBU0523 had an unknown Fimtype. Antimicrobial resistance was observed in three isolates (11%, 3/27). Among these, one isolate exhibited resistance to quinolones, specifically ciprofloxacin and nalidixic acid, while one isolate was resistant to folate pathway antagonists (sulfamethoxazole), and one isolate harboured resistance to macrolides, specifically azithromycin (Table 2).

**TABLE 2:** Genetic characteristics of 27 *Escherichia coli* isolates from the Jukskei River.

Isolate	Phylogroups	MLST	Serotype	*fimH* type	Antibiotic resistance
ECBR0223	A	8759	O16:H48	*fimH2521*	None
ECBR0423	A	6083	O16:H12	*fimH305*	None
ECBR0723	A	8107	O16:H25	*fimH27*	Quinolone (Ciprofloxacin and nalidixic acid)
ECBR1023	A	8765	O16:H48	*fimH305*	None
ECBR1123	A	889	O16:H48	*fimH27*	None
ECGI0223	A	2968	O16:H48	*fimH27*	None
ECGI0423	B1	13,526	O37	*fimH121*	None
ECGI0523	A	10,333	O16:H48	*fimH27*	None
ECGI0723	A	10,410	O16:H48	*fimH2*	None
ECGI1023	B2	3439	O16:H1	*fimH10*	None
ECGI1123	B2	13,039	O16	*fimH580*	None
ECMA0223	A	5074	O16:H48	*fimH27*	None
ECMA0423	B1	12,434	O13:H14	*fimH34*	Macrolides (azithromycin)
ECMA0523	A	5247	O16:H25	*fimH27*	None
ECMA0723	A	6070	O16:H48	*fimH27*	None
ECMA1123	B1	889	O16:H25	*fimH32*	None
ECBU0223	B1	3957	O16:H48	*fimH27*	None
ECBU0423	B1	6640	O16:H48	*fimH34*	None
ECBU0523	A	2025	O16:H48	—	None
ECBU0723	B1	7088	O16:H48	*fimH34*	None
ECBU1023	A	6402	O16:H48	*fimH27*	None
ECBU1123	A	9616	O16	*fimH27*	None
ECDA0223	A	—	O16:H48	*fimH27*	None
ECDA0423	A	3553	O16:H48	*fimH27*	None
ECDA0723	A	1224	O16:H48	*fimH27*	None
ECDA1023	A	5295	O16	*fimH27*	Folate pathway antagonist (sulfamethoxazole)
ECDA1123	A	8668	O16:H48	*fimH27*	None

*Note:* —, not detected.

**FIGURE 2:**
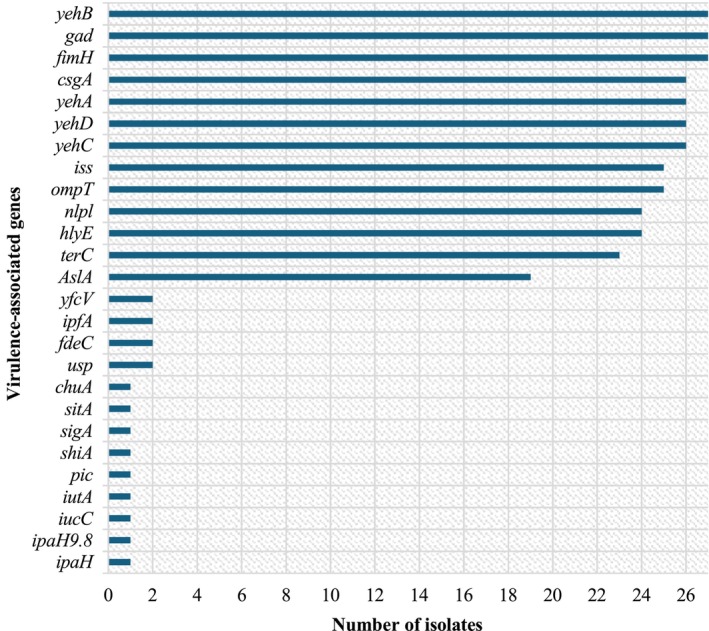
Frequency of VAGs in the 27 *Escherichia coli* isolates from the Jukskei River.

A total of 26 VAGs were detected among the 27 *E. coli* isolates. These included adherence‐associated genes (*fimH, csgA, fdeC, ipfA, yfcV, yehA, yehB, yehC* and *yehD*), invasion‐associated genes (*ompT, nlpl, AslA, iss*), stress response *gad* and *terC* genes, nutrition and metabolism‐associated genes (*chuA, iucC, iutA, sitA*), as well as toxin‐associated genes (*usp, hlyE, ipaH, ipaH9.8, pic, shiA* and *sigA*). The gene pattern *fimH*‐*gad*‐*csgA*‐*iss*‐*ompT*‐*yehA‐D* was frequently observed in more than 90% of the isolates (*n* ≥ 25), with the most common VAGs being *fimH*, *gad* and *yehB*, occurring in 100% (27/27) of the isolates. Whereas the *hlyE* and *nlpl* genes were detected in 89% (24/27) of the isolates, while the *terC* and *AslA* genes were detected at 85% (23/27) and 70% (19/27) of the isolates, respectively. The *usp*, *fdeC* and *ipfA* VAGs were detected in only 7% (2/27) (Figure 2). Notably, one isolate (ECMA0423) possessed additional genes including *ipaH*, *ipaH9.8*, *iucC*, *pic*, *iut, AshiA*, *sigA* and *sitA*, which are linked to enhanced pathogenic potential. This isolate was initially classified as an ETEC based on the pathotype‐specific PCR and is now classified as a hybrid ETEC/EIEC pathotype.

### 3.1 Molecular Characterisation of *E. coli* Isolates

#### 3.1.1 Pathotypes Multiplex PCR

Genomic DNA was extracted from a loopful of *E. coli* colonies grown overnight on 5% blood agar (Thermo Fisher Scientific, USA) using the QIAamp DNA Mini Kit (Qiagen, GmbH, Germany). DNA concentration and purity (between 10 and 100 ng/μL) were assessed using a NanoDrop 2000 spectrophotometer (Thermo Fisher Scientific, MA, USA). Five separate PCR assays (A–E) targeting various pathotype‐specific virulence‐associated genes (VAGs) were performed using primer sequences adapted from Alfinete et al. (2022) (Table 3). The assays were optimised using known positive controls obtained from the National Institute for Communicable Diseases (NICD). All PCR reactions were performed in an Applied Biosystems MiniAmp Thermal Cycler (Thermo Fisher Scientific, MA, USA) in a final volume of 25 μL. Reactions A and B contained 12.5 μL of 2× PCR master mix (Thermo Fisher Scientific, MA, USA), 0.5 μL of each primer, 7.5 μL of nuclease‐free water and 2 μL of genomic DNA (10–100 ng/ μL). Reactions C and D included 12.5 μL of 2× PCR master mix (Thermo Fisher Scientific, MA, USA), 0.5 μL of each primer, 8.5 μL of nuclease‐free water and 2 μL of genomic DNA. For assay E, targeting the *lt1* gene, the reaction mixture consisted of 12.5 μL of 2× PCR master mix (Thermo Fisher Scientific, MA, USA), 2 μL of each primer, 6.5 μL of nuclease‐free water and 2 μL of genomic DNA. Thermal cycling conditions for all assays were as follows: an initial activation step at 95°C for 15 min; 35 cycles of denaturation at 94°C for 45 s, annealing at 54°C for 45 s and extension at 68°C for 2 min; followed by a final extension at 72°C for 5 min. Positive controls and a NTC were included in each run. The PCR amplicons were resolved on a 2% (w/v) agarose gel with 10 μL of GelRed (Thermo Fisher Scientific, MA, USA) and electrophoresed at 110 V for 45 min. A 100 bp molecular weight marker (Anatech, South Africa) was used to estimate amplicon sizes.

**TABLE 3:** Primer sequences used in the amplification of different pathotype‐specific VAGs in *Escherichia coli* .

Pathovar	Target gene	Primer sequence (5′→3′)	Product size (bp)	Multiplex PCR reaction	References
EHEC	*eaeA*	F: CTG AAC GGC GAT TAC GCG AA R: CCA GAC GAT ACG ATC CAG	917	A	(Aranda et al. 2004)
	*stx1*	F: ACA CTG GAT GAT CTC AGT GG R: CTG AAT CCC CCT CCA TTA TG	614	A	(Omar and Barnard 2014)
	*stx2*	F: CCA TGA CAA CGG ACA GCA GTT R: CCT GTC AAC TGA GCA CTT TG	779	A	(Omar and Barnard 2014)
EIEC	*ial*	F: GGT ATG ATG ATG ATG AGT CCA R: GGA GGC CAA CAA TTA TTT CC	650	B	(López‐Saucedo et al. 2003)
EAEC	*eagg*	F: AGA CTC TGG CGA AAG ACT GTA TC R: ATG GCT GTC TGT AAT AGA TGA GAA C	194	B	(Pass et al. 2000)
*E. coli* toxin	*asta*	F: CCA TGA CAA CGG ACA GCA GTT R: CCT GTC AAC TGA GCA CTT TG	106	B	(Kimata et al. 2005)
Atypical EPEC	*eaeA*	F: CTG AAC GGC GAT TAC GCG AA R: CCA GAC GAT ACG ATC CAG	917	C	(Aranda et al. 2004)
Typical EPEC	*bfpA*	F: AAT GGT GCT TGC GCT TGC TGC R: TAT TAA CAC CGT AGC CTT TCG CTG AAG TAC CT	410	C	(Omar and Barnard 2014)
EHEC of serogroup O157	*rfbE*	F: CGG ACA TCC ATG TGA TAT GG R: TTG CCT ATG TAC AGC TAA TCC	259	D	
EHEC of serogroup O111	*wbdI*	F: TAG AGA AAT TAT CAA GTT AGT TCC R: ATA GTT ATG AAC ATC TTG TTT AGC	406	D	
ETEC	*lt1*	F: TGG ATT CAT CAT GCA CCA CAA GG R: CCA TTT CTC TTT TGC CTG CCA TC	360	E	(Pass et al. 2000)

Abbreviations: *asta*, enteroaggregative thermostable enterotoxin (EAST1 toxin); *bfpA*, bundle‐forming pilus; *eaeA*, intimin outer membrane protein; *eagg*, antiaggregative protein; *ial*, invasion‐associated locus; *lt1*, heat‐labile toxin; *rfbE*, part of O‐antigen 157; *stx*, Shiga toxin; *wbdI*, part of O‐antigen 111.

An isolate was classified as enterohemorrhagic *E. coli* (EHEC) if positive for *eaeA* and either *stx1* or *stx2*. It was EIEC if positive for *ial*. The isolate was EAEC if positive for *eagg*. The isolate was atypical enteropathogenic *E. coli* (aEPEC) if positive for *eaeA* alone, and as typical EPEC (tEPEC) if positive for both *eaeA* and *bfpA*. Isolates were further identified as EHEC of serogroup O157 or O111 if positive for *rfbE* or *wbdI*, respectively. ETEC was identified if positive for *lt1*. The presence of the *asta* gene confirmed the presence of the *E. coli* heat‐stable enterotoxin. The gene targets and corresponding primer sequences used for PCR are summarised in Table 3.

#### 3.1.2 Whole Genome Sequencing

To conduct WGS, genomic DNA was extracted from a loopful of *E. coli* colonies grown overnight on 5% blood agar (Thermo Fisher Scientific, USA) using the QIAmp DNA Kit (Qiagen, GmbH, Germany). The concentrations of genomic DNA were determined using the Qubit dsDNA assay HS (High Sensitivity) Assay Kit (Thermo Fisher, MA, USA). The DNA libraries were prepared using the Rapid Barcoding Kit 24 V14 (SQK114.24) (Oxford Nanopore) and loaded into the MInION flow cell (Oxford Nanopore) following the manufacturer's protocol. Sequencing reactions were performed on the MinKNOW Mk1B platform using the MinKNOW Software 23.11.2 (Oxford Nanopore). Reference‐based genome assemblies were constructed from the raw reads using the bacterial genome workflow on the EPI2ME software 5.1.10. The reference genome ( *E. coli* strain K‐12 substrain MG1655, accession number: U00096.3) used to map the raw reads was retrieved from the *National Center for Biotechnology Information (NCBI)* (https://www.ncbi.nlm.nih.gov/datasets/genome/). The genome sequences were uploaded to NCBI under the accession number: PRJNA1187560.

#### 3.1.3 Bioinformatics Analysis

Species verification of the assembled genomes was performed using SpeciesFinder 2.0 (https://cge.food.dtu.dk/services/SpeciesFinder/) (Larsen et al. 2014). The assembled genomes were used to identify the sequence types (STs) using the MLST 2.0.9 program (https://cge.food.dtu.dk/services/MLST/). The serotypes were determined using SerotypeFinder 2.0 software (https://cge.food.dtu.dk/services/SerotypeFinder/) for *E. coli* . FimTyper 1.0 (https://cge.food.dtu.dk/services/FimTyper/) (Roer et al. 2017) was used to predict the *E. coli* Fim types. Identification of acquired antibiotic‐resistance genes (ARGs) within the genomes of *E. coli* was performed using ResFinder 4.4.3 software (https://cge.food.dtu.dk/services/ResFinder/) (Camacho et al. 2009; Bortolaia et al. 2020), and VAGs were determined using the VirulenceFinder 2.0.5 software (https://cge.food.dtu.dk/services/VirulenceFinder/) (Joensen et al. 2014; Malberg Tetzschner et al. 2020).

#### 3.1.4 Phylogenetic Analysis

Phylogroups were assigned for each isolate using Clermont Typing and genetic profile classification (Beghain et al. 2018) accessed on http://clermontyping.iame‐research.center/. In cases where the Clermont Typing result differed from the Mash group assignment, the Mash group was prioritised. For isolates with an unknown phylogroup from Clermont Typing, the Mash group classification was used instead.

The genetic relationship between the isolates collected from the Jukskei River and those from other sources was examined. A total of 11 *E. coli* genomes available from EnteroBase (https://enterobase.warwick.ac.ul/) were selected for phylogenetic analysis. The search criteria used on EnteroBase were ‘ *E. coli*’, ‘South Africa’ and ‘2023’. A total of 11 genomes matched our search criteria. Of these, nine genomes were from humans and two from livestock. Whole‐genome sequences of all isolates were uploaded and analysed on the CSI Phylogeny 1.4 pipeline (https://cge.cbs.dtu.dk/services/CSIPhylogeny/). The maximum likelihood single nucleotide polymorphism (SNP) tree was constructed using CSI Phylogeny 1.4 (call SNPs and infer phylogeny) of Centre for Genomic Epidemiology with a default input parameter: minimum depth at SNP positions; 10, relative depth at SNP positions; 10, minimum distance between SNPs (prune); 10, minimum SNP quality; 30, minimum read mapping quality; 25, minimum Z‐score; 1.96 was applied for maximum likelihood phylogeny tree construction (Kaas et al. 2014). The *E. coli* strain K‐12 substrain MG1655, accession number: U00096.3, was used as the reference genome. The constructed tree was visualised and annotated using Interactive Tree of Life (iTOL) (Letunic and Bork 2024).

### 3.2 Phylogenetic Analysis

**FIGURE 3:**
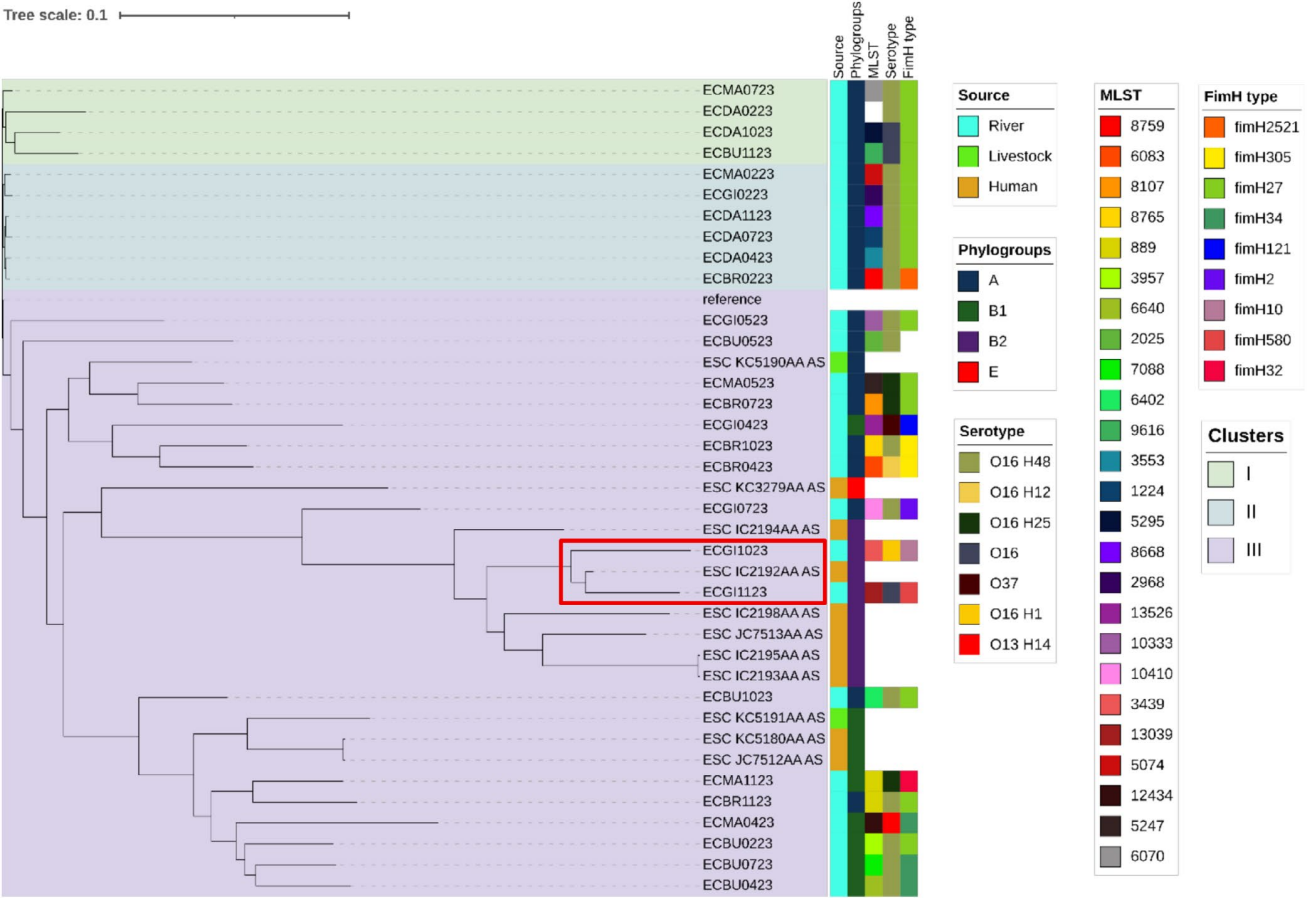
An SNP‐based maximum likelihood Phylogenetic Tree. The tree represents the phylogenetic relationships among the different isolates based on the SNP data obtained from the genomes using *Escherichia coli* K‐12 MG1655 as a reference genome. The annotations on the tips of the tree provide information about each isolate, including the isolate source (river, human and livestock), phylogroups, MLST, serotypes and FimH types. The tree is presented with branch lengths to emphasise relationships. Cluster I, II and III, are highlighted using light colours (green, blue and purple).

Phylogroup A dominated the collection at 70% (19/27), followed by B1 at 22% (6/27) and then B2 (7%, 2/27). The SNP‐based maximum likelihood clustering of genomes grouped the isolates into three clusters (I, II and III) on the phylogenetic tree (Figure 3). Isolates from the Jukskei River were spread across the different clusters, reflecting genomic diversity among *E. coli* isolates. Clusters I and II contain phylogroup A isolates from rivers, whereas cluster III, the largest cluster, had isolates from the river as well as human and livestock origin. Specifically, five isolates from the Jukskei River (ECBR0423, ECBR1023, ECBR0723, ECGI0423 and ECMA0523) formed a monophyletic relationship with isolate ESC_KC5190AA_AS from livestock, suggesting that these strains descended from a single common ancestor. Notably, these isolates belong to phylogroup A, except isolate ECGI0423, which was assigned to phylogroup B1 by both Mash group assignment and Clermont Typing. Another monophyletic relationship was observed between six isolates from the Jukskei River (ECMA1123, ECBR1123, ECMA0423, ECBU0223, ECBU0723, ECBU0423), one isolate from livestock (ESC_KC5191AA_AS) and two isolates from human origin (ESC_KC5180AA_AS and ESC_JC7512AA_AS). Notably, these isolates belong to phylogroup B1, except for ECBR1123, which was assigned to phylogroup A based on Mash group assignment, which differed from the Clermont Typing (phylogroup B1). Based on the clustering on the phylogenetic tree, this isolate likely belongs to phylogroup B1. Interestingly, a close monophyletic relationship between two river isolates (ECGI1023 and ECGI1123) and an isolate ESC_IC2192AA_AS from human origin was observed.

## 4 Discussion

DEC are an important public health concern among children in low‐ and middle‐income countries like South Africa. These infections are primarily associated with the consumption of contaminated food and water, with DEC particularly linked to severe gastrointestinal diseases (Croxen et al. 2013; Gomes et al. 2016; Abey et al. 2024). In our study, 44% of the isolates were classified as DEC, including ETEC, aEPEC and a hybrid ETEC/EIEC isolate (identified through WGS). The EPEC and ETEC pathotypes have been reported as the predominant pathotypes in many South African rivers and water bodies (Abia et al. 2017; Bolukaoto et al. 2021). Delair et al. (2024) reported EPEC and ETEC as the most prevalent pathotypes in recreational water bodies in the Gauteng Province of South Africa. Moreover, EPEC was the most common pathotype in the rivers surrounding the Vhembe district in Limpopo Province (Potgieter et al. 2020). Further highlighting the widespread presence of these pathotypes in South African surface waters. In contrast to our findings, ETEC was not detected in the Jukskei River in a 2021 study by Hoorzook et al. (2021). Whereas in our study, the majority of the DEC detected from the Jukskei River were ETEC. This may suggest a shift in the microbial profile of the Jukskei River to resemble that of other developing countries as one of the sampling sites was near an informal settlement, which is home to several foreign nationals from the Southern African Developing Communities (SADC) as well as other African countries that often seek asylum and better opportunities (Ekanade and Molapo 2017). Therefore, some of the ETEC cases are likely imported from other African countries. In the African continent, ETEC is predominant in the surface waters in Nigeria (Titilawo et al. 2015) and Côte d'Ivoire (Kambire et al. 2017). Moreover, the ETEC pathotype has also been detected in South African wells and borehole water in peri‐urban communities (Abia et al. 2017). Similarly, in India and Bolivia, ETEC was the most widespread pathotype in surface waters and rivers (Abia et al. 2017; Bolukaoto et al. 2021). Nevertheless, the presence of EPEC and ETEC in the Jukskei River is concerning, as these pathotypes are among the leading etiological agents of childhood diarrhoea in developed countries (Nataro et al. 2006; Kotloff et al. 2013), and are associated with severe diseases and outbreaks (Park et al. 2018; Buuck et al. 2020; Lim et al. 2020).

To gain a deeper insight into the genetic diversity of the *E. coli* isolates from the Jukskei River, WGS was employed as it provides comprehensive information on the genomic content of microorganisms (Uelze et al. 2020). The isolates in our study exhibited high ST diversity based on Achtman's 7‐gene MLST scheme; however, the majority of the isolates were serotype O16:H48. The *E. coli* O16:H48 is a subset of the nonpathogenic *E. coli* K12 MG1655 strain that is widely used in laboratories and is a commensal of the human gut (Lang et al. 2018; Lee et al. 2021). Interestingly, a study carried out by Bergeron et al. (2012) isolated extraintestinal pathogenic *E. coli* strains containing the O16:H48 serotype in abattoirs in 2005 and 2007 in Canada. In 2005, one strain was susceptible to the common antimicrobials, whereas in 2007, another strain was resistant to streptomycin, sulfisoxazole and tetracycline. This further highlights *E. coli* K12 as a continuously evolving human gut commensal (Bhat et al. 2019). Notably, fimbrial typing revealed nine Fimtypes among the 27 *E. coli* isolates, with *fimH27* being the most prevalent type. Fimbriae are extracellular structures that facilitate bacterial adhesion to host tissues, playing a crucial role in colonisation and establishment of infection (Kline et al. 2009; Gonyar et al. 2024). In China, *fimH27* was the prevalent fimtype in *E. coli* isolated from patients with community‐onset bloodstream infections (Chen et al. 2021), whereas a study in Mozambique reported an emerging *E. coli* ST131‐*fimH27* subclone responsible for bacteremia in Mozambican children. Moreover, this subclone exhibited a hybrid pathotype, carrying both enterotoxigenic and enteroaggregative fimbriae genes (Mandomando et al. 2020).

The majority of the *E. coli* isolates in our study carried no ARGs, except for three isolates that showed resistance to ciprofloxacin, sulfamethoxazole and azithromycin antibiotics commonly used to treat certain enteric *E. coli* , such as ETEC, particularly in cases of traveller's diarrhoea (CDC 2024). Although antimicrobial resistance does not appear to be a major concern among these river isolates, the presence of multiple VAGs, however, indicates that virulence remains a significant public health concern. In total, 26 VAGs were detected among the 27 *E. coli* isolates. These VAGs were categorised into five classes, including adherence, invasion, stress survival, nutrition and metabolism as well as exotoxins. The majority of VAGs were associated with adherence and exotoxins, with six of the toxin‐associated genes occurring in one isolate. These included *csgA, fimH, gad, hlyE, iss, nlpl, ompT, yehA‐D*. Similar VAGs have been reported in environmental *E. coli* isolates obtained from a wastewater treatment plant and its receiving waters in KwaZulu‐Natal, South Africa (Mbanga et al. 2021). Other VAGs of interest detected in our study were *AslA*, *terC*, *usp*, *fdeC*, *ipfA* and *yfcV*. The *AslA* gene was detected in 70% of isolates and is responsible for the invasion of the brain by crossing the blood–brain barrier, a mechanism specific to neonatal meningitis *E. coli* (Kim 2001; Hammad et al. 2022). The *terC* gene was present in 85% of *E. coli* isolates and is a part of the *ter* operon that confers tellurite resistance (Valková et al. 2007). The *usp* gene is a precursor of the protein responsible for genotoxin activity in mammalian cells, causing bacteremia (Rihtar et al. 2020). The *fdeC* gene is a nonfimbrial intimin‐like adhesin (Aleksandrowicz et al. 2021). The *ipfA* gene is essential for adherence and colonisation of the intestine by encoding long fimbriae (Mahazu et al. 2021). The y*fcV* gene was present in 3% of the isolates and encodes a major subunit of chaperone‐usher fimbriae (Mahazu et al. 2021).

Interestingly, one *E. coli* isolate harboured the *ipaH*, *ipaH*9.8, *shiA*, *sigA* and *sitA* genes, respectively. These VAGs are those of EIEC and are similar to those found in *Shigella* following the successful acquisition of the pINV and horizontal gene transfer of genes such as *shiA*, *sigA* and *sitA*. The *ipaH* gene is essential for the successful invasion of host cells, specifically *Shigella* and EIEC (Zhang et al. 2021). The *ShiA* gene is found predominantly in the pathogenicity island *Shigella* island 2 (SHI‐2) and facilitates iron uptake and immune response evasion by regulating the activities of proteins involved in these processes (Pasqua et al. 2017). The *sigA* gene encodes cytotoxins responsible for the accumulation of intestinal fluid in infected hosts (Pakbin et al. 2021). The *sitA* gene facilitates iron uptake in *Shigella* (Runyen‐Janecky et al. 2003). The ECMA0423 isolate was thus classified as an EIEC, an etiological agent of bacillary dysentery in humans, particularly in developing countries. Concerningly, the EIEC pathotype was missed by the pathotype PCR assay, possibly due to a mutation in the primer binding site of the target gene; however, the isolate was positive for the *lt*1 gene for an ETEC, suggesting a hybrid pathotype. Moreover, this isolate carried resistance to azithromycin. Hybrid *E. coli* pathotypes are not unusual and have been reported in the environment (Bolukaoto et al. 2021) as well as in clinical settings (Alfinete et al. 2022) and in livestock (Awad et al. 2020; Ogunbiyi et al. 2023). Specifically, a South African study by Bolukaoto et al. (2021) reported an alarming presence of hybrid pathotypes in three rivers in Gauteng, including the Jukskei River.

Three phylogroups, A, B1 and B2, were identified among the *E. coli* isolates from the Jukskei River, with phylogroup A being the most dominant, followed by B1 and then B2. According to Clermont et al. (2013), phylogroup A is primarily from commensal lineages and is commonly associated with humans. However, some members of this group have evolved into pathogenic strains, such as EAEC and ETEC (Chekole et al. 2025). Phylogroup A has also been reported as the most prevalent phylogroup in South African studies, particularly in fresh produce (Plessis et al. 2017) and pig farms (Strasheim et al. 2024). Similarly, in Portugal, phylogroups A and B1 were the most prevalent in irrigation water and vegetables, suggesting a pattern of environmental and agricultural prevalence (Araújo et al. 2017). Phylogroup B1 is typically considered an environmental lineage, often associated with livestock. However, it has also been implicated in human extraintestinal infections, including sepsis (Reid et al. 2020). Notably, some B1 strains are known to persist in water environments, which could explain their presence in the Jukskei River (Berthe et al. 2013). The predominance of phylogroup A among our *E. coli* isolates suggests that the river is primarily contaminated with faecal matter of human origin, and the presence of B1 and B2 further indicates mixed contamination sources, including livestock and environmental reservoirs. Moreover, this phylogroup distribution highlights potential public health risks, as several of these phylogroups are associated with both intestinal and extraintestinal infections (Kelly et al. 2024). The evolutionary relationships of 27 *E. coli* isolates from the Jukskei River and 11 isolates from human and livestock sources were further analysed using phylogenetic methods. The river isolates were dispersed across multiple branches of the phylogenetic tree, indicating high genetic diversity, likely due to multiple contamination sources such as runoff, sewage and animal waste. Furthermore, evidence of potential transmission between livestock, river water and human sources was observed in Cluster III, where isolates from different origins clustered together, suggesting shared ancestry. A close monophyletic relationship between the five river isolates and a livestock isolate was observed, possibly indicating the role of animals in contaminating the river. Another close monophyletic relationship was found between two river isolates and an isolate from human origin. These findings highlight the Jukskei River as a potential reservoir and vehicle for *E. coli* transmission and emphasise the importance of integrated surveillance across environmental, human and animal sectors.

A limitation of this study is the relatively small number of *E. coli* isolates analysed, which may not fully capture the genetic diversity and population structure of the *E. coli* strains present in the Jukskie River. Future studies should include a larger sample size to provide a more comprehensive understanding of the genetic diversity in this environment. Moreover, epidemiological sampling of surrounding communities and livestock is needed to better understand the role of the river in transmitting DEC.

## 5 Conclusion

To the best of our knowledge, this is the first WGS study of a collection of *E. coli* from the Jukskei River, and our findings highlight this river as a reservoir for DEC that has the potential to cause severe diarrheal diseases. In previous studies conducted at the Jukskei River, EPEC was the prevalent pathotype, while ETEC was predominant in our study, suggesting a possible shift in the microbial profile of the Jukskei River. Phylogenetic analysis revealed a greater phylogenetic spread among *E. coli* isolates from the river, reflecting environmental heterogeneity and diverse contamination sources (e.g., runoff, sewage, animal). Moreover, a clonal relationship was also observed between some isolates from the river and isolates from human and livestock origin, highlighting possible cross‐environmental transmission, emphasising the importance of integrated surveillance across environmental, human and animal sectors.

## Author Contributions

**Luyanda Mkhize:** investigation, writing – original draft, methodology, visualization, writing – review and editing, validation, software, project administration. **Musa Marimani:** supervision, resources, writing – review and editing, funding acquisition, validation, investigation, software. **Sanelisiwe Thinasonke Duze:** conceptualization, investigation, funding acquisition, writing – review and editing, supervision, resources, project administration, validation.

## Ethics Statement

This study was approved by the Human Research Ethics Committee (Medical) of the University of the Witwatersrand and an ethical waiver (W‐CBP‐220530‐02) was provided.

## Consent

The authors have nothing to report.

## Conflicts of Interest

The authors declare no conflicts of interest.

## Data Availability

The data that support the findings of this study are available from the corresponding author upon reasonable request.
